# Prevalence and influencing factors of insomnia in patients with coronary heart disease: a systematic review and meta-analysis

**DOI:** 10.3389/fpsyt.2026.1748293

**Published:** 2026-04-01

**Authors:** Shu-lan Liu, Ya Chen, Xiao-di Bai, Ting Xu, He-yao Xu, Si-yu Lin, Xin-yao Zhou, Yun-lan Jiang

**Affiliations:** 1 College of Nursing, Chengdu University of Traditional Chinese Medicine, Chengdu, China; 2 Hospital of Chengdu University of Traditional Chinese Medicine, Chengdu, China

**Keywords:** coronary heart disease, insomnia, meta-analysis, prevalence, risk factors

## Abstract

**Background:**

Sleep is a pivotal component of cardiovascular health. However, clinical efforts in coronary heart disease (CHD) disproportionately focus on sleep-disordered breathing, while insomnia, the most prevalent sleep disorder among adults, remains underdiagnosed and undertreated.

**Aims:**

This meta-analysis systematically evaluates the prevalence of insomnia and its influencing factors in patients with CHD to inform clinical prevention and management strategies.

**Methods:**

Ten databases were searched from inception to 7 September 2025. The prevalence rates, odds ratios (ORs), and 95% confidence intervals (CIs) were extracted to evaluate the prevalence of insomnia and its influencing factors in patients with CHD. RevMan 5.4 and Stata 15.0 software was used for data processing. Subgroup analyses, meta-regression, and sensitivity analyses were performed.

**Results:**

Nineteen studies involving 5928 patients with CHD were included. The overall pooled prevalence of insomnia was 51.8% (95% CI: 0.446–0.590, *P* < 0.001). Significant risk factors identified were female sex(OR = 2.00, 95% CI: 1.58–2.52, *P* < 0.001), anxiety (OR = 1.61, 95% CI: 1.36–1.91, *P* < 0.001), depression (OR = 2.15, 95% CI: 1.48–3.13, *P* < 0.001), CHD duration ≥3 years (OR = 1.73, 95% CI: 1.25–2.40, *P* = 0.001), diabetes (OR = 1.50, 95% CI: 1.45–1.56, *P* < 0.001), and gastritis (OR = 2.24, 95% CI: 1.62–3.11, *P* < 0.001).

**Conclusion:**

insomnia has a substantial prevalence in patients with CHD. Clinicians should prioritize early identification and intervention targeting modifiable risk factors (anxiety, depression, diabetes, and gastritis), particularly in female patients and those with a CHD duration ≥3 years.

**Systematic review registration:**

https://www.crd.york.ac.uk/prospero/, identifier CRD42024617785.

## 1 Introduction

Coronary heart disease (CHD), an atherosclerosis-driven disease characterized by luminal narrowing and insufficient myocardial perfusion, poses a continuing challenge as a leading cause of global morbidity and mortality (1–3). As cardiovascular prevention and treatment research advances, the recent recognition of sleep health as a critical indicator of cardiovascular wellness has brought the impact of sleep disorders into sharp focus (4). Insomnia is the most common sleep disorder, defined as a sleep continuity disturbance associated with daytime complaints and characterized by difficulty initiating sleep, difficulty maintaining sleep, or early morning awakening (5). According to the American Heart Association’s 2025 report, insomnia is closely linked to the prognosis of cardiovascular disease, doubling office visits, increasing emergency room visits by 40%, and contributing an estimated $94.9 billion in annual costs (6).

The relationship between insomnia and CHD is complex and bidirectional (4). In one direction, CHD precipitates insomnia through concomitant sympathetic activation, systemic inflammation, disease-related somatic symptoms, and psychological distress (7–9). Reciprocally, insomnia further activates the sympathetic nervous system and the hypothalamic-pituitary-adrenal (HPA) axis, triggering hemodynamic changes, metabolic disturbances, and the release of inflammatory cytokines. These mechanisms collectively impair endothelial function, thereby accelerating coronary atherosclerosis and increasing the risk of adverse cardiovascular events (10–12). Moreover, clinical evidence demonstrates a dose-response relationship, whereby poorer sleep quality in CHD patients is associated with a correspondingly higher all-cause mortality risk (13). This intricate pathophysiological interplay underscores the need to recognize and manage insomnia as an integral component of CHD care (14).

Although several studies have been conducted in recent years to investigate the association between insomnia and CHD, the true burden of insomnia within the CHD population remains poorly defined. The reported prevalence rates of insomnia vary considerably across studies (15), and the evidence regarding whether the association between insomnia and CHD is influenced by age, sex, or other demographic characteristics remains limited and controversial (16). These divergent findings hinder clinical consensus, likely limited to methodological variations, such as the insomnia assessment tool and study design (15, 17). Therefore, this study performed a systematic review and meta-analysis to quantitatively estimate the pooled prevalence of insomnia in patients with CHD and its influencing factors, thereby providing an evidence base to guide early recognition and clinical management.

## 2 Methods

### 2.1 Design

This study protocol was registered on the International Prospective Register of Systematic Reviews (PROSPERO) platform (No. CRD42024617785) and was conducted following the Preferred Reporting Items for Systematic Reviews and Meta-Analyses (PRISMA) guidelines (18).

### 2.2 Selection criteria

Inclusion criteria: (I) population: adult patients (age ≥18 years) with a confirmed diagnosis of CHD; (II) exposure: diagnosis or assessment of insomnia; (III)outcomes: the prevalence of insomnia and/or its associated influencing factors, with data provided as prevalence, odds ratios (ORs) with 95% confidence intervals (CIs), or convertible raw data; (IV) study design: observational studies, including cross-sectional, case-control, and cohort designs; (V) language: no restrictions on publication language.

Exclusion criteria:(I) studies including participants in a cardiac intensive care unit (CCU) setting or with diagnosed sleep-breathing disorders; (II)studies that employed tools with a lack of objective validation for insomnia diagnosis; (III)conference abstracts, case reports, reviews, letters, or commentaries; (IV) duplicate publication, full text not available, data not available, or low-quality literature.

### 2.3 Data sources and search strategies

PubMed, Embase, Web of Science, Cochrane Library, PsycINFO, CINAHL, SinoMed, CNKI, Wanfang, and VIP Database were searched from inception to 7 September 2025. Medical subject headings (MeSH) terms were combined with free words to search, and references included in the study were traced back if necessary. Detailed search strategies are provided in **Supplementary File 1**, with the PubMed search strategy shown below as an example: (“Coronary Artery Disease”[MeSH Terms] OR “Coronary Disease”[MeSH Terms] OR “angina, stable”[MeSH Terms] OR “angina, unstable”[MeSH Terms] OR “Acute Coronary Syndrome”[MeSH Terms] OR (“coronary heart disease”[Title/Abstract] OR “coronary atherosclerotic heart disease”[Title/Abstract] OR “ischemic heart disease”[Title/Abstract] OR “angina pectoris”[Title/Abstract] OR “myocardial ischemic”[Title/Abstract] OR “myocardial infarct*”[Title/Abstract])) AND (“Sleep Initiation and Maintenance Disorders”[MeSH Terms] OR (“insomnia”[Title/Abstract] OR “dyssomnia”[Title/Abstract] OR “sleeplessness”[Title/Abstract] OR “sleep dysfunction”[Title/Abstract] OR “sleep disorder*”[Title/Abstract] OR “sleep quality”[Title/Abstract])) AND (“risk factor*”[Title/Abstract] OR “related factor*”[Title/Abstract] OR “relevant factor*”[Title/Abstract] OR “influen*”[Title/Abstract] OR “correlat*”[Title/Abstract] OR “associat*”[Title/Abstract] OR “predict*”[Title/Abstract]).

### 2.4 Study selection and data extraction

Studies were managed using EndNote 21. Two researchers (L.S.L. and B.X.D.) independently screened titles and abstracts, followed by full-text assessment, based on predefined inclusion and exclusion criteria. The screening results were subsequently cross-compared, and conflicts were settled through consultation with a third researcher (X.H.Y.). A pre-designed Excel template was used to extract: title, first author, publication year, country, sample source, sample size, study design, participant age, prevalence rates, influencing factors, effect sizes (OR, 95% CI), and diagnostic instrument.

### 2.5 Quality assessment

Two researchers (L.S.L. and C.Y.) independently assessed the methodological quality of the included studies. The Newcastle-Ottawa Scale (19) was used to assess cohort and case-control studies, evaluating selection (0–4 points), comparability (0–2 points), and outcome (0–3 points) for a maximum score of 9. Based on this scoring system, studies were classified as low (0–4 points), moderate (5–6 points), or high (7–9 points) quality. The Agency for Healthcare Research and Quality (20) tool was used for cross-sectional studies and consists of 11 items, each rated as “Yes” (1 point), “No” (0 points), or “Unclear” (0 points). Based on the total AHRQ scores, studies were categorized as low (0–3 points), moderate (4–7 points), or high (8–11 points) quality. Disagreements were resolved through discussion or by consulting a third researcher (X.T.).

### 2.6 Data synthesis and analysis

Data analysis was performed using Stata 15.0 and RevMan 5.4. Pooled prevalence and ORs with 95% CIs were calculated. Heterogeneity across the included studies was assessed using the *I*² statistic and Cochran’s *Q* test. A random-effects model was used if *I*² ≥ 50% and *P* < 0.10; otherwise, a fixed-effects model was applied (*P* ≥ 0.10 and *I*² < 50%). Sensitivity analyses were conducted using the leave-one-out method and comparing the pooled estimates under fixed-effect and random-effects assumptions. Subgroup analyses and meta-regression based on prespecified study characteristics were performed to explore potential causes of heterogeneity. Finally, publication bias was examined by visual inspection of funnel plot symmetry and quantified using Egger’s and Begg’s tests.

## 3 Results

### 3.1 Search results

**Figure 1:**
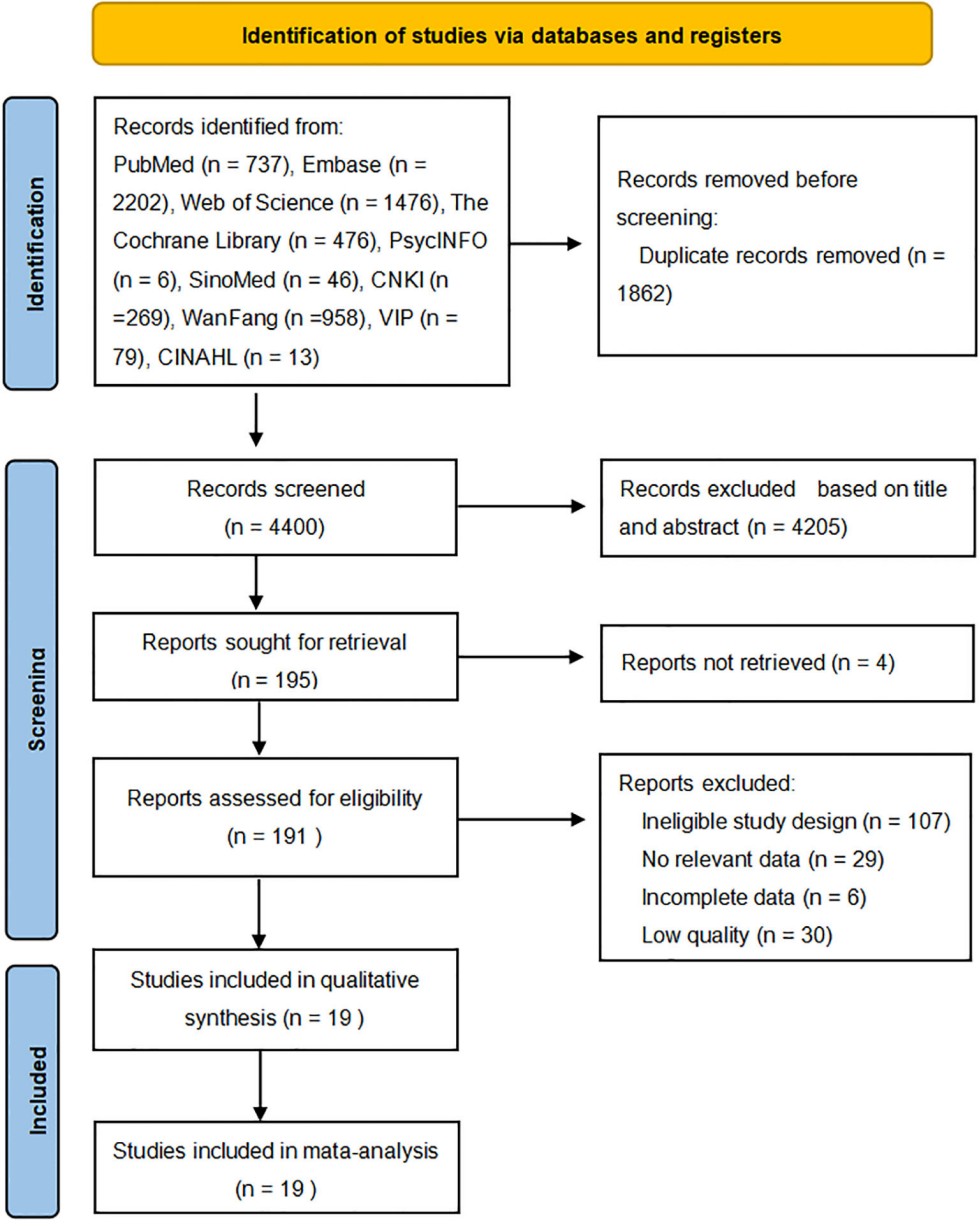
Flow diagram of the searching and screening.

A systematic search of ten electronic databases yielded 6,262 records. After removing 1,862 duplicates, 4,205 records were excluded based on title and abstract screening. Finally, 19 studies were retained after assessing the full texts of 195 articles, of which 4 articles could not be retrieved and 172 were excluded (see **Figure 1**).

### 3.2 Characteristics of included studies

This study incorporated 19 studies (1 case-control, 4 cohort, and 14 cross-sectional) spanning 8 countries and involving 5,928 cases (**Table 1**). Most studies (15/19) were published within the past five years, and all demonstrated moderate to high methodological quality (see **Supplementary File 2**).

**Table 1:** Characteristics of included studies.

Study	Publication year	Country	Study design	Sample source	Age: mean ± SD	Sample size (male)	Prevalence(%)	Influencing factors	Diagnostic criteria	Quality rating
Coryel (65)	2013	America	cross-sectional	A	58.3 ± 10.6	102 (59)	37.25%	Anxiety, depression, liver disease	ISI	M
Costa (66)	2017	Canada	cross-sectional	B	64.8 ± 11.7	209 (152)	35.89%	Depression, age, greater dysfunctional beliefs about sleep, prescribed sleep medications	ISI	H
Ning (67)	2019	China	cross-sectional	A	73.85 ± 10.04	169 (75)	71.88%	Anxiety, depression, age, prescribed sleep medications, respiratory diseases, sex, hypertension	PSQI	M
Muthukrishnan (68)	2020	India	prospective cohort study	A	55.6 ± 12.05	187 (151)	66.84%	Anxiety, BMI>30, diabetes, sedentary activity	PSQI	H
Wu (69)	2021	China	cross-sectional	B	—	501 (356)	37.92%	Sex, gastritis, CHD duration ≥3 years, URRBMI, Canadian Cardiovascular Society class >I	PSQI	M
Frøjd (70)	2021	Norway	cross-sectional	B	62 ± 10	1082 (855)	45.10%	Anxiety, depression, age, sex, diabetes, CRP ≥2mmol/L, eating fish<3 times/week, type D personality	BIS	H
Zhang (21)	2021	China	cross-sectional	A	—	110 (49)	67.27%	Age, sex, living arrangement, exercise	PSQI	M
Kong (71)	2021	China	prospective cohort study	B	60.5 ± 12.0	741 (510)	65.32%	Depression, pre-admission physical activity level, low social support	DSM-5	H
Cheng (72)	2022	China	cross-sectional	A	62.6 ± 11.2	348(272)	47.13%	Anxiety, depression	PSQI	M
Gökçe (73)	2023	Turkey	cross-sectional	A	66.50 ± 10.32	60(27)	83.33%	Additional chronic diseases coexisting with CHD	PSQI	M
Zheng (9)	2023	China	cross-sectional	A	61.58 ± 6.93	240(126)	46.67%	Anxiety, depression, age, sex, type of health insurance	PSQI	M
Yang (74)	2024	China	cross-sectional	A	68.14 ± 11.72	244 (141)	58.61%	Anxiety, depression, diabetes, gastritis	PSQI	M
He (75)	2024	China	cross-sectional	A	62.46 ± 8.94	515 (408)	46.41%	Age, the score on the abstraction domain of the MoCA	PSQI	M
Huang (76)	2024	China	retrospective cohort study	A	29-95	256 (207)	35.16%	Anxiety, depression, age, smoke, NYHA, unfamiliar environment	PSQI	H
Yakut (77)	2024	Turkey	prospective cohort study	A	60.37 ± 12.23	424 (333)	41.51%	Age, diabetes, SYNTAX score>22, incomplete revascularization	PSQI	M
Mo (78)	2025	China	cross-sectional	A	—	345 (255)	20.87%	Anxiety, depression, age, sex, gastritis, CHD duration ≥3 years	PSQI	M
Hbaieb (79)	2025	Tunisia	cross-sectional	A	56.77 ± 8.24	60 (60)	33.33%	—	PSQI	M
Najfath (80)	2025	India	cross-sectional	A	58.59 ± 9.47	150 (96)	72.67%	—	PSQI	M
Wassif (81)	2025	Egypt	case-control	A	55.0 ± 7.0	185 (89)	86.49%	—	PSQI	M

A, Single-center; B, Multi-center; H, High quality; M, Moderate quality; ISI, Insomnia Severity Index; PSQI, Pittsburgh Sleep Quality Index; DSM-5, Diagnostic and Statistical Manual of Mental Disorders, Fifth Edition; BIS, the Bergen Insomnia Scale; BMI, Body Mass Index; URRBMI, Urban and Rural Resident Basic Medical Insurance; CRP, C-reactive Protein; MoCA, the Montreal Cognitive Assessment; NYHA, New York Heart Function Assessment (NYHA) class; SYNTAX, The SYNergy between percutaneous intervention with TAXus drug-eluting stents and cardiac surgery.

### 3.3 Prevalence of insomnia in patients with CHD

**Figure 2:**
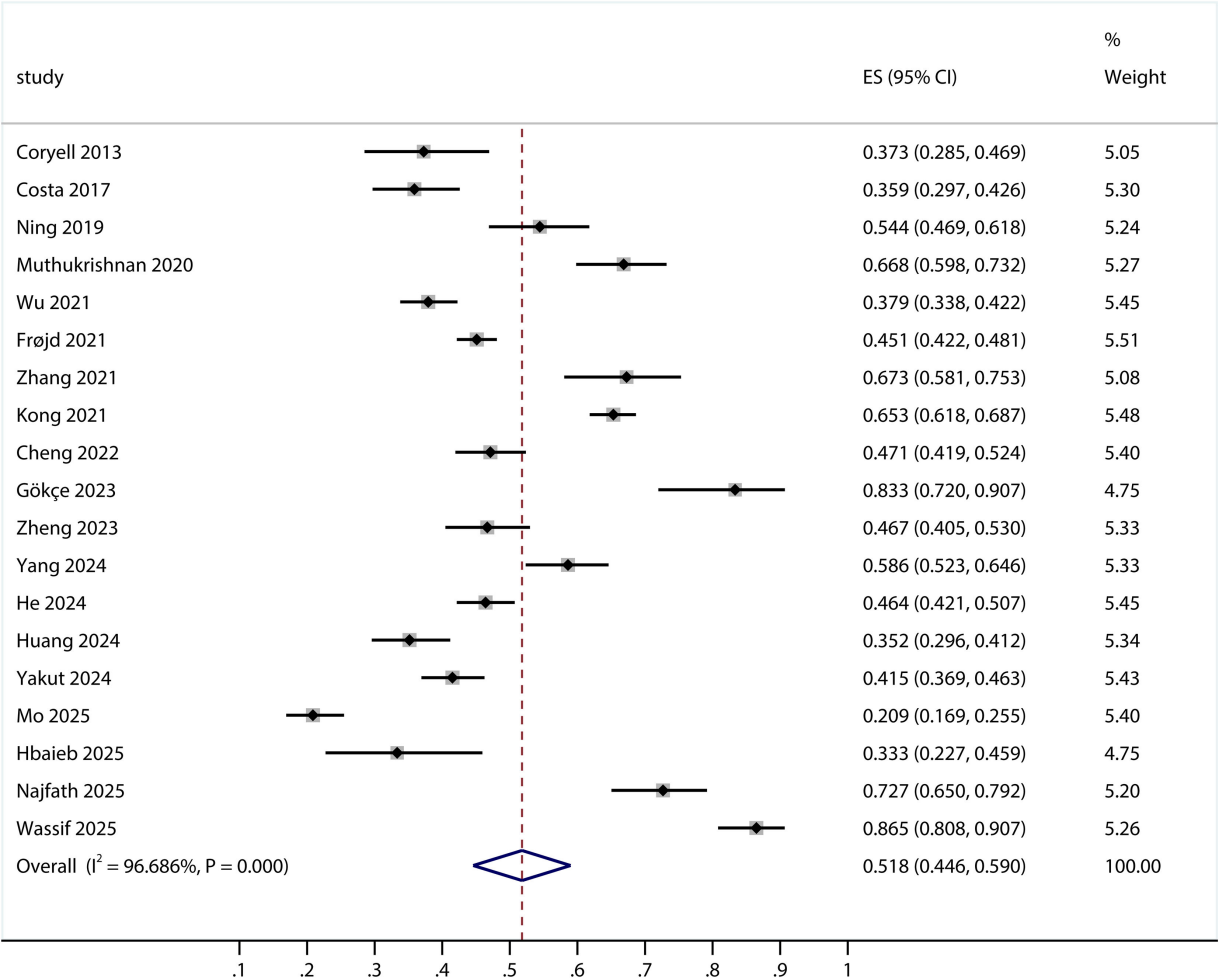
Forest plot of incidence of insomnia in patients with coronary heart disease.

All 19 included studies reported the prevalence of insomnia in patients with CHD, with rates ranging from 20.87% to 86.49%. Due to significant heterogeneity (*I*² = 96.686%, *P* < 0.001), a random-effects model was employed, yielding a pooled overall prevalence of 51.8% (95% CI: 44.6%–59.0%, *P* < 0.001; **Figure 2**), with the prediction interval ranging from 16% to 87% (**Supplementary File 3**).

### 3.4 Subgroup analysis and meta-regression analysis

Subgroup analysis revealed the potential sources of heterogeneity (**Table 2**). Demographically, a higher prevalence of insomnia was observed in females (57.6%) compared to males (45.5%). While age, marital status, and educational level had no significant effect on the combined effect size (*P* for interaction > 0.05). Regarding clinical factors, patients without physical activity had a significantly higher insomnia prevalence (79.5%) compared to those who engaged in physical activity (50.4%). In contrast, the type of CHD, BMI, smoking history, and drinking history had no significant effect on the prevalence of insomnia (*P* for interaction > 0.05). Regarding comorbidities, insomnia was closely associated with psychological conditions, with prevalence rates reaching 72.9% and 77.8% for comorbid anxiety and depression, whereas the prevalence of insomnia among patients with comorbid hypertension, diabetes, and hyperlipidemia was 49.1%, 51.1%, and 42.6%. Regarding methodological factors, area, study design, and diagnostic instrument were the sources of heterogeneity. Specifically, the prevalence of insomnia was highest in Europe (75.6%), followed by Asia (52.9%), Africa (45.1%), and North America (36.3%); the prevalence in case-control studies (86.5%) was higher than in cohort studies (52.3%) and cross-sectional studies (48.7%); studies using the DSM-5 reported higher prevalence rates (65.3%) than those using the PSQI (53.4%), BIS (45.1%), and ISI (36.3%), while publication year had no significant effect on the pooled estimates (*P* for interaction > 0.05).

**Table 2:** Subgroup analyses of the pooled prevalence of insomnia in patients with coronary heart disease.

Subgroup	Number of studies	Sample size	Heterogeneity		Effect model	Pooled prevalence (95%CI)	*P*-interaction
			*I* ^2^	*P*			
Demographic factors							
Sex							0.031
Male	13	3430	95.698%	<0.001	Random	0.455(0.371,0.539)	
Female	13	1342	84.316%	<0.001	Random	0.576(0.506,0.645)	
Age							0.167
≥60	4	580	92.987%	<0.001	Random	0.613(0.445,0.769)	
<60	3	458	—	—	Random	0.438(0.263,0.621)	
Marital status							0.324
Married/Cohabiting	7	2053	96.333%	<0.001	Random	0.488(0.370,0.608)	
Unmarried/Widowed/Divorced	5	179	82.032%	<0.001	Random	0.600(0.414,0.773)	
Education							0.525
High school and below	9	2583	94.191%	<0.001	Random	0.461(0.377,0.545)	
College and above	9	846	75.744%	<0.001	Random	0.423(0.346,0.501)	
Clinical factors							
Types of CHD							0.139
ACS	7	1838	95.455%	<0.001	Random	0.427(0.316,0.543)	
CCS	4	733	96.765%	0.014	Random	0.604(0.401,0.790)	
BMI							0.298
≥24	6	1066	91.765%	<0.001	Random	0.466(0.352,0.582)	
<24	3	527	— —	—	Random	0.353(0.191,0.535)	
Physical activity							0.006
Yes	4	1570	94.252%	<0.001	Random	0.504(0.379,0.629)	
No	4	513	93.268%	<0.001	Random	0.795(0.630,0.922)	
Smoking history							0.781
Yes	11	1372	91.600%	<0.001	Random	0.491(0.393,0.589)	
No	11	1804	95.559%	<0.001	Random	0.512(0.400,0.624)	
Drinking history							0.942
Yes	4	484	94.917%	<0.001	Random	0.424(0.221,0.641)	
No	3	577	—	—	Random	0.436(0.208,0.680)	
Co-existing diseases							<0.001
Hyperlipidemia	8	699	88.968%	<0.001	Random	0.426(0.294,0.563)	
Hypertension	9	1812	94.626%	<0.001	Random	0.491(0.389,0.593)	
Diabetes	9	907	92.375%	<0.001	Random	0.511(0.391,0.630)	
Anxiety	5	569	92.115%	<0.001	Random	0.729(0.584,0.854)	
Depression	5	406	85.608%	<0.001	Random	0.778(0.654,0.881)	
Methodological factors							
Publication year							0.144
2013-2019	3	480	—	—	Random	0.425(0.306,0.549)	
2020-2025	16	5448	97.116%	<0.001	Random	0.535(0.455,0.615)	
Area							<0.001
North America	2	311	—	—	Random	0.363(0.310,0.418)	
Asia	14	4290	96.593%	<0.001	Random	0.529(0.446,0.611)	
Europe	2	245	—	—	Random	0.756(0.699,0.808)	
Africa	1	1082	—	—	Random	0.451(0.422,0.481)	
Study design							<0.001
Cross-sectional	14	4135	94.862%	<0.001	Random	0.487(0.417,0.558)	
Cohort	4	1608	97.411%	<0.001	Random	0.523(0.364,0.679)	
Case-control	1	185	—	—	Random	0.865(0.808,0.907)	
Diagnostic instrument							<0.001
PSQI	15	3794	96.769%	<0.001	Random	0.534(0.443,0.623)	
ISI	2	311	—	—	Random	0.363(0.310,0.418)	
DSM-5	1	741	—	—	Random	0.653(0.618,0.687)	
BIS	1	1082	—	—	Random	0.451(0.422,0.481)	

Meta-regression (using area, publication year, sample size, study design, and diagnostic instrument as covariates) identified no significant sources of heterogeneity (*P* > 0.05 for all; **Supplementary File 4**). Nevertheless, the limited number of studies might compromise the statistical power, precluding the exclusion of potential sources.

### 3.5 Risk factors of insomnia in patients with CHD

Meta-analysis of factors reported in ≥2 studies identified six significant risk factors for insomnia in patients with CHD (**Table 3**): female sex, anxiety, depression, CHD duration ≥3 years, diabetes, and gastritis.

**Table 3:** Pooled risk factors of insomnia in patients with coronary heart disease.

Risk factors	Number of studies	Sample size	Pooled effects		Effect model	Heterogeneity	
			OR(95%CI)	*P*		*I* ^2^	*P*
Age	8	3141	1.02 (0.97,1.06)	0.51	Random	81%	<0.001
Female sex	5	2337	2.00 (1.58,2.52)	<0.001	Fixed	0%	0.88
Anxiety	9	2973	1.61 (1.36,1.91)	<0.001	Random	91%	<0.001
Depression	9	3527	2.15 (1.48,3.13)	<0.001	Random	82%	<0.001
CHD duration ≥3 years	2	846	1.73 (1.25,2.40)	0.001	Fixed	0%	0.35
Diabetes	4	1937	1.50 (1.45,1.56)	<0.001	Fixed	0%	0.54
Gastritis	3	1090	2.24 (1.62,3.11)	<0.001	Fixed	0%	0.50

### 3.6 Sensitivity analysis and publication bias

**Figure 3:**
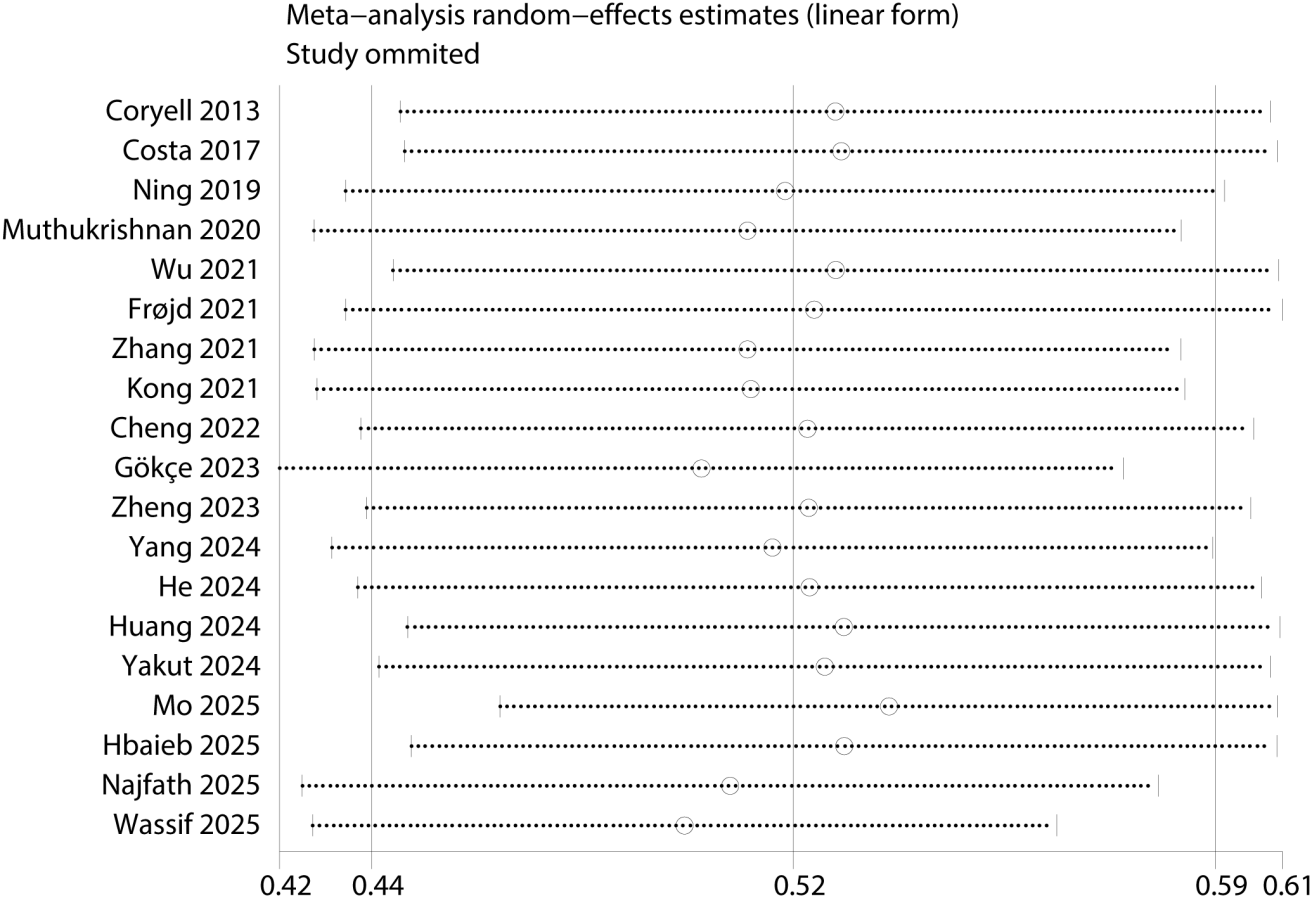
Sensitivity analysis of the pooled prevalence of insomnia in patients with coronary heart disease.

**Figure 4:**
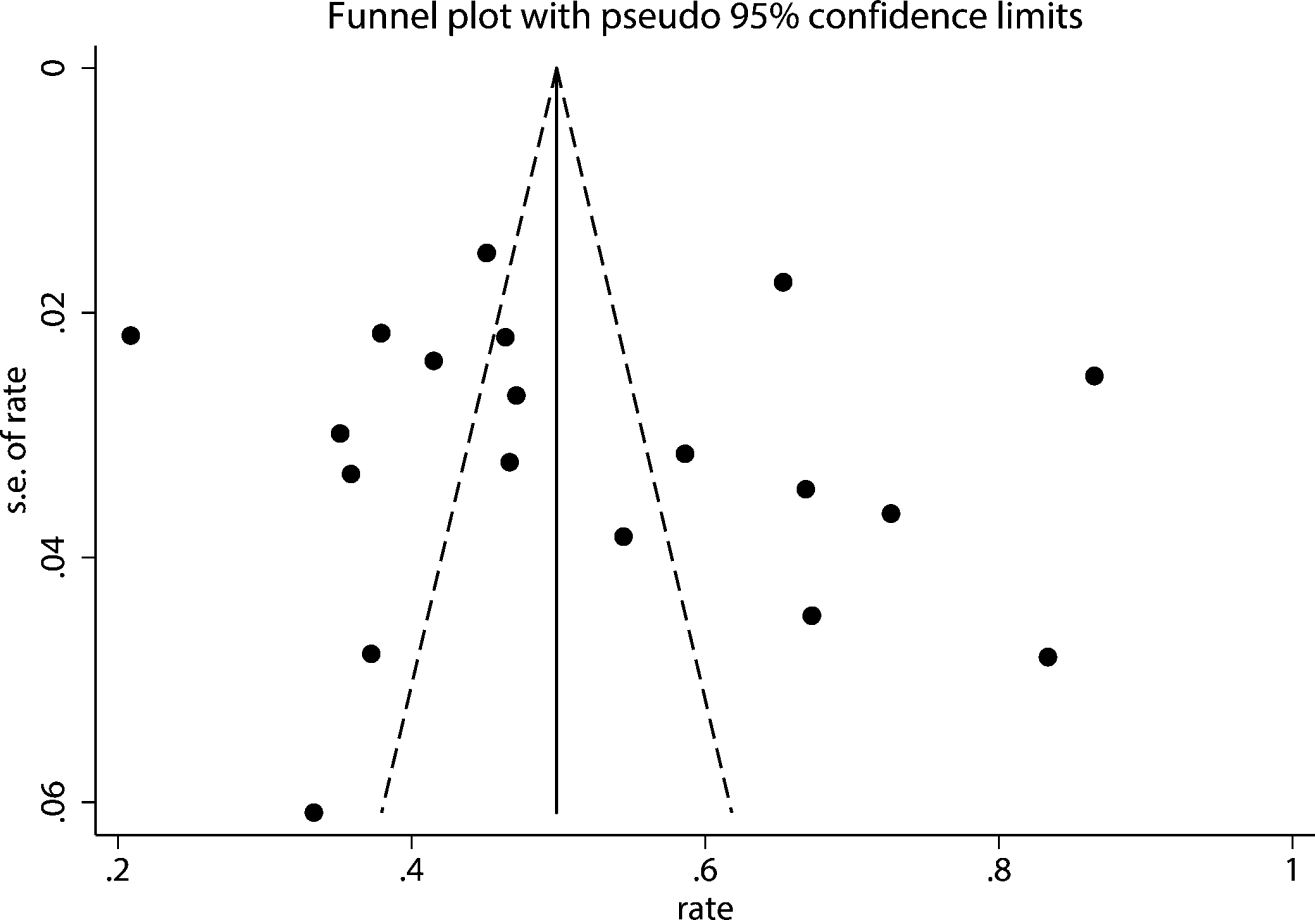
Publication bias in the prevalence of insomnia in patients with coronary heart disease.

Leave-one-out sensitivity analysis of prevalence estimates revealed no substantial changes (**Figure 3**). However, pronounced instability was observed in the analysis of female sex as a influencing factor (*I*² = 98%, OR = 1.39, 95% CI: 0.43–4.52, *P* = 0.59). After excluding the study by Zhang et al. (21), heterogeneity was resolved (*I*² = 0%), and the association became significant (OR = 2.00, 95% CI: 1.58–2.52, *P* < 0.001). This identifies this study as a key source of heterogeneity, potentially because it exclusively enrolled elderly patients. Furthermore, the high concordance between the pooled effect estimates from the random-effects and fixed-effects models further supports the stability of all significant associations (**Table 4**). Finally, no significant publication bias was detected based on a symmetric funnel plot (**Figure 4**) and non-significant results from Egger’s (*P* = 0.545) and Begg’s tests (*P* = 0.208).

**Table 4:** Sensitivity analysis of risk factors for insomnia in patients with coronary heart disease.

Influencing factors	Random effect model			Fixed effects model			Stability
	OR	95%CI	*P*	OR	95%CI	*P*	
Age	1.02	0.97-1.06	<0.001	1.00	0.99-1.02	<0.001	Stable
Female sex	2.00	1.58-2.52	<0.001	2.00	1.58-2.52	<0.001	Stable
Anxiety	1.61	1.36-1.91	<0.001	1.19	1.16-1.21	<0.001	Stable
Depression	2.15	1.48-3.13	<0.001	1.18	1.11-1.25	<0.001	Stable
CHD duration of ≥3 years	1.73	1.25-2.40	0.001	1.73	1.25-2.40	0.001	Stable
Diabetes	1.50	1.45-1.56	<0.001	1.50	1.45-1.56	<0.001	Stable
Gastritis	2.24	1.62-3.11	<0.001	2.24	1.62-3.11	<0.001	Stable

## 4 Discussion

Insomnia is a known psychosocial factor influencing the prognosis of CHD, yet its prevalence and associated risk factors in this population have not been systematically quantified. In this meta-analysis of 19 studies (N = 5,928), we provide the first comprehensive assessment, revealing a pooled insomnia prevalence of 51.8% and identifying several significant risk factors: female sex, anxiety, depression, CHD duration ≥3 years, diabetes, and gastritis.

### 4.1 Prevalence of insomnia in CHD patients

The findings indicate that approximately half of patients with CHD are affected by insomnia, suggesting that insomnia has become a clinical issue that cannot be overlooked in the comprehensive management of CHD. However, there might be obvious heterogeneity, as evidenced by the wide prediction interval (16%–87%). This pooled estimate should be regarded as a reference range for the overall epidemiological trend, rather than a precise estimate applicable to any clinical setting.

Subgroup analyses revealed important variations in insomnia prevalence among patients with CHD. The higher prevalence in females compared to males warrants attention (22, 23). Previous studies have shown that women have higher levels of inflammatory mediators and exhibit a stronger cortisol response to stress (24); hormonal fluctuations during key reproductive phases, such as pregnancy and menopause, may further exacerbate this phenomenon (25). These findings support the implementation of targeted preventive measures for insomnia in women, particularly during these phases. Furthermore, sex differences extend to sleep patterns. A study reported that CHD was associated with longer sleep duration in men and shorter sleep duration in women (26). In contrast, a large multicenter cohort study found that a 7-hour sleep duration was associated with the lowest mortality risk for both sexes, and sleeping less than 5 hours confers greater cardiovascular mortality risk for men (27). Collectively, these nuanced findings underscore the importance of thoroughly considering the combined effects of sex, age, physiological stage, and sleep patterns in the management of CHD.

Regarding clinical factors, lack of physical activity was associated with a higher prevalence of insomnia, which is consistent with previous findings (28). Current evidence suggests that 4 to 12 minutes of moderate-to-vigorous physical activity during the day can offer significant cardiometabolic health benefits (6); excessive exercise may increase the risk of insomnia and adverse cardiovascular events (28). However, the optimal intensity of physical activity for patients with CHD remains inconclusive (13), and widespread kinesiophobia poses a challenge to exercise strategies (29, 30). This represents a current research frontier in formulating personalized exercise programs that balance cardiovascular safety with sleep quality through comprehensive assessments of multiple individual factors.

Psychological comorbidities (anxiety 72.9%, depression 77.8%) showed substantially stronger associations with a higher incidence of insomnia compared to traditional cardiometabolic comorbidities (hypertension 49.1%, hyperlipidemia 42.6%, diabetes 51.1%), consistent with a prior study (31). However, the ranking of cardiometabolic comorbidities by insomnia prevalence in our study differs from that reported in a previous study focused exclusively on patients with ACS (32, 33). This discrepancy suggests that the influence of specific comorbidities on sleep may differ across CHD subtypes and warrants targeted investigation.

Analysis of heterogeneity across methodological factors yielded several findings. Geographically, the prevalence of insomnia among patients with CHD is the highest in Europe (75.6%), followed by Asia (52.9%), Africa (45.1%), and North America (36.3%). While factors such as climate, sociocultural norms, and healthcare standards may partly explain (34, 35), this finding warrants cautious interpretation, as most included studies originated in Asia, with only one from Africa and two each from Europe and North America. This observation underscores a critical gap in global research and highlights the need for future multicenter studies to validate this difference. Regarding study design, cross-sectional studies reported lower insomnia prevalence rates than cohort or case-control studies, suggesting potential underdiagnosis in shorter-term assessments and underscoring the value of long-term follow-up for accurate evaluation. Future research could employ prospective cohort studies to explore the trajectories of insomnia and their association with the progression of CHD. We noted that studies using the DSM-5 reported a higher prevalence of insomnia, likely reflecting its greater sensitivity. It is worth noting that While Self-report scales remain the predominant assessment tool in clinical and large-scale studies owing to their cost-effectiveness and convenience relative to polysomnography, their inherent limitations, such as reporting and recall bias, must be recognized. A critical priority for future research is the cost-effective integration of objective and subjective measures to establish standardized insomnia screening pathways that facilitate a comprehensive assessment of sleep health in patients with CHD (36).

### 4.2 Risk factors of insomnia in CHD patients

Identified risk factors offer crucial targets for prevention. Our analysis revealed female sex as a significant risk factor, whereas age showed no independent association. Although previous studies generally suggest that sleep duration decreases with age (37, 38), this aging-related effect (e.g., cortical thinning, white matter degeneration, neurotransmitter dysregulation, and circadian rhythm disruptions) may be masked by the profound impact of CHD (e.g., myocardial ischemia, medication side effects) (39). This may explain why the age factor was not significant in our study. Additionally, women experienced significantly more severe and referred pain than men (40), potentially elevating their insomnia risk and reinforcing observed sex-based differences. Notably, insomnia not only exacerbates cardiovascular impairment but also accelerates cognitive decline (41). However, clinical interventions face significant challenges. A Shortage of professionals limits the first-line cognitive-behavioral therapy (CBT), while potential side effects constrain pharmacotherapy. More critically, existing treatments are plagued by significant interindividual variability in efficacy and a lack of standardized protocols. This underscores the urgent need for personalized therapeutic approaches and enhanced training for primary care providers (42). Encouragingly, aerobic exercise has garnered widespread attention for its positive effects on cognitive function, mental health, and sleep quality (43). And structured physical activity is recommended as part of comprehensive management plans for patients with chronic diseases to mitigate medication side effects and age-related functional decline (44). Future research could further explore comprehensive intervention plans focused on exercise therapy for managing insomnia in CHD patients across different age groups.

This study confirmed that anxiety and depression are independent predictors of insomnia in patients with CHD, consistent with previous research (45). Neuroimaging studies on insomnia disorder consistently have further shown overlapping (salience network: insula and anterior cingulate cortex) and differential MRI correlation patterns between depressive (thalamus, orbitofrontal cortex and its associated functional connectivity) and anxiety (functional connectivity associated with default mode network) symptoms (46). Psychological distress affects 15%–40% of CHD patients, disrupts sleep architecture, and is linked to hypercoagulability, inflammation, and immune dysfunction, but less attention has been paid to mental health in clinical practice (47, 48). Psychological interventions improve anxiety and depression symptoms but fail to reduce mortality or major adverse cardiovascular events (48, 49). Additionally, escitalopram’s superiority over exercise for alleviating anxiety and depression symptoms (50), highlights the limitations of non-pharmacological approaches. Therefore, identifying and addressing psychological issues in CHD patients remains a challenge that needs to be addressed. Concurrently, as a current hotspot, healthy diet patterns have demonstrated positive effects in mental health by regulating factors such as blood sugar, immunity, and gut microbiota, underscoring the potential value of nutritional interventions (51).

Diabetes emerged as another risk factor for insomnia in patients with CHD (52). Potential contributors to this observed association include sleep disruption from nocturia, neuropathic pain, glycemic fluctuations, and psychological distress (53, 54). This comorbidity presents a therapeutic challenge, yet novel antidiabetic agents such as SGLT-2 inhibitors and GLP-1 receptor agonists have substantially improved glycemic control in this population (55, 56). Additionally, nutritional strategies offer significant benefits. For instance, the Mediterranean diet has been shown to improve lipid profiles, blood pressure, endothelial function, and insulin resistance while providing superior renal protection (57). Likewise, magnesium and zinc supplementation improve glycemic control, enhance antioxidant and anti-inflammatory capacity, and ameliorate anxiety and depression (58). Specifically, with advancing genomic and proteomic research in this field, future research will further elucidate mechanisms and identify novel therapeutic targets for precision medicine (59, 60).

Furthermore, gastritis is associated with insomnia in CHD patients, although the underlying mechanisms remain unclear. Prior research suggests that gut microbiota may modulate sleep homeostasis via the circulation or the vagus nerve by metabolizing neuroactive substances (e.g., serotonin, GABA) and short-chain fatty acids (e.g., butyrate) (61, 62), offering a potential theoretical perspective for understanding the link between gastrointestinal and sleep disorders. Accordingly, gut microbiota-targeted interventions, such as probiotic supplementation and increased dietary fiber intake, have been explored as adjunctive strategies to improve sleep (63, 64). However, given that the present study primarily relied on observational evidence, a causal relationship cannot be established, and the specific mechanisms warrant validation in high-quality prospective studies. Future analysis of the gut microbiota in CHD patients may help identify novel diagnostic and therapeutic targets (62).

Finally, our study identified a CHD duration of ≥3 years as a significant risk factor for insomnia. Although the rationale for this specific cutoff is unclear, a prolonged disease course is typically associated with more severe chronic myocardial ischemia, persistent systemic inflammation, and an accumulated psychological burden. This underscores the need for greater clinical attention to sleep disturbances in this long-duration subgroup and warrants future research to establish an evidence-based framework for disease-duration stratification. Such efforts are essential for elucidating the progression and pathophysiology of insomnia risk and for guiding precise management strategies.

## 5 Limitations and strengths

This study benefits from a rigorous methodology following PRISMA/PROSPERO, comprehensive subgroup and sensitivity analyses, and the identification of key risk factors across demographic, clinical, and psychosocial domains, which help screen high-risk patients and provide a basis for formulating targeted prevention strategies.

However, several limitations should be acknowledged. First, despite addressing heterogeneity through random-effects models and exploring it via subgroup analyses/metaregression/sensitivity analyses, the prevalence estimates may still be affected by differences in diagnostic instruments, insufficient reporting of clinical variables related to CHD (such as disease duration, comorbidities, and medication use), and the diversity of study designs. Second, all studies included in the present analysis employed observational designs (mainly cross-sectional), which precludes causal inference. Accordingly, future prospective studies are needed to further validate these findings. Third, the exclusion of CCU-based studies limits the application of our conclusions to this particular unit. Fourth, insufficient data precluded a more detailed stratified analysis of CHD subtypes; the specific prevalence rates of insomnia across different subtypes remain to be determined. Finally, the relatively high proportion of Asian populations included in the study may indicate insufficient attention to insomnia issues among CHD patients in other regions, and also limit the generalizability of these findings.

## 6 Conclusion

This meta-analysis, for the first time, comprehensively pools the overall prevalence of insomnia (51.8%) in CHD patients, indicating that clinical attention to sleep problems needs to be strengthened. It is recommended that structured insomnia assessment training be provided to nursing staff to promote the standardization and normalization of sleep assessment in cardiovascular care settings. Six key risk factors were identified, including female sex, anxiety, depression, CHD duration ≥ 3 years, diabetes, and gastritis. Despite the possible confounding effects of disease burden and medication use, the above findings still have clinical reference value, suggesting that early identification and intervention of anxiety and depression should be emphasized, especially in patients with longer disease duration or coexisting diabetes and gastritis. In the future, large-scale, multi-center prospective studies are necessary to further clarify the association mechanism and development trajectory between insomnia and CHD, so as to develop more effective, personalized management strategies.

## Data Availability

The original contributions presented in the study are included in the article/**Supplementary Material**. Further inquiries can be directed to the corresponding author.
