# Supplementary material for: Prevalence and influencing factors of insomnia in patients with coronary heart disease: a systematic review and meta-analysis
Source: Front Psychiatry. 2026 Apr 1;17:1748293. doi: 10.3389/fpsyt.2026.1748293 (PMC13079287; doi:10.3389/fpsyt.2026.1748293)
Supplement: Supplementary file 1 [file Supplementaryfile1.docx]

**Supplementary materials**

**contents page：**

Supplementary File 1. Search strategy for ten databases

Supplementary File 2: Quality assessment

Supplementary File 3: Prediction interval

Supplementary File 4: Meta-regression analysis results

**Supplementary File 1: Search strategy for ten databases**

| **1.PubMed: 737 Results** |
| --- |
| #1 "Coronary Artery Disease"[MeSH Terms] OR "Coronary Disease"[MeSH Terms] OR "angina, stable"[MeSH Terms] OR "angina, unstable"[MeSH Terms] OR "Acute Coronary Syndrome"[MeSH Terms] |
| #2 "coronary heart disease"[Title/Abstract] OR "coronary atherosclerotic heart disease"[Title/Abstract] OR "ischemic heart disease"[Title/Abstract] OR "angina pectoris"[Title/Abstract] OR "myocardial ischemic"[Title/Abstract] OR "myocardial infarct*"[Title/Abstract] |
| #3 #1 OR #2 |
| #4 "Sleep Initiation and Maintenance Disorders"[MeSH Terms] |
| #5 "insomnia"[Title/Abstract] OR "dyssomnia"[Title/Abstract] OR "sleeplessness"[Title/Abstract] OR "sleep dysfunction"[Title/Abstract] OR "sleep disorder*"[Title/Abstract] OR "sleep quality"[Title/Abstract] |
| #6 #4 OR #5 |
| #7 "risk factor*"[Title/Abstract] OR "related factor*"[Title/Abstract] OR "relevant factor*"[Title/Abstract] OR "influen*"[Title/Abstract] OR "correlat*"[Title/Abstract] OR "associat*"[Title/Abstract] OR "predict*"[Title/Abstract] |
| #8 #3 AND #6 AND #7 |
| **2. Embase: 2202 Results** |
| #1 'coronary artery disease'/exp |
| #2 'coronary disease':ab,ti OR 'angina,stable':ab,ti OR 'angina, unstable':ab,ti OR 'acute coronary syndrome':ab,ti OR 'coronary heart disease':ab,ti OR 'coronary atherosclerotic heart disease':ab,ti OR 'ischemic heart disease':ab,ti OR 'angina pectoris':ab,ti OR 'myocardial ischemic':ab,ti OR 'myocardial infarct*':ab,ti |
| #3 #1 OR#2 |
| #4 'insomnia'/exp |
| #5 'sleep initiation and maintenance disorders':ab,ti OR 'dyssomnia':ab,ti OR 'sleeplessness':ab,ti OR 'sleep dysfunction':ab,ti OR 'sleep disorder*':ab,ti OR 'sleep quality':ab,ti |
| #6 #4 OR #5 |
| #7 'risk factor*':ab,ti OR 'related factor*':ab,ti OR 'relevant factor*':ab,ti OR 'influen*':ab,ti OR 'correlat*':ab,ti OR 'associat*':ab,ti OR 'predict*':ab,ti |
| #8 #3 AND #6 AND #7 |
| **3. Web of Science: 1476 Results** |
| #1 TS=(“coronary artery disease” OR “coronary disease” OR “angina,stable” OR “angina, unstable” OR “acute coronary syndrome” OR “coronary heart disease” OR “coronary atherosclerotic heart disease” OR “ischemic heart disease” OR “angina pectoris” OR “myocardial ischemic” OR “myocardial infarct*”) |
| #2 TS=(“sleep initiation and maintenance disorders” OR “insomnia” OR “dyssomnia” OR “sleeplessness” OR “sleep dysfunction” OR “sleep disorder*” OR “sleep quality”) |
| #3 TS=(“risk factor*” OR “related factor*” OR “relevant factor*” OR “influen*” OR “correlat*” OR “associat*” OR “predict*”) |
| #4 #1 AND #2 AND #3 |
| **4. Cochrane Library: 476 Results** |
| #1 MeSH descriptor: [Coronary Artery Disease] explode all trees |
| #2 (coronary disease):ab,ti,kw OR (angina,stable):ab,ti,kw OR (angina, unstable):ab,ti,kw OR (acute coronary syndrome):ab,ti,kw OR (coronary heart disease):ab,ti,kw OR (coronary atherosclerotic heart disease):ab,ti,kw OR (ischemic heart disease):ab,ti,kw OR (angina pectoris):ab,ti,kw OR (myocardial ischemic):ab,ti,kw OR (myocardial infarct*):ab,ti,kw |
| #3 #1 OR #2 |
| #4 MeSH descriptor: [Sleep Initiation and Maintenance Disorders] explode all trees |
| #5 (insomnia):ab,ti,kw OR (sleeplessness):ab,ti,kw OR (sleep dysfunction):ab,ti,kw OR (sleep disorder*):ab,ti,kw OR (sleep quality):ab,ti,kw |
| #6 #4 OR #5 |
| #7 (risk factor*):ab,ti,kw OR (related factor*):ab,ti,kw OR (relevant factor*):ab,ti,kw OR (influen*):ab,ti,kw OR (correlat*):ab,ti,kw OR (associat*):ab,ti,kw OR (predict*):ab,ti,kw |
| #8 #3 AND #6 AND #7 |
| **5.PsycINFO: 6 Results** |
| Title: “coronary artery disease” OR “coronary disease” OR “angina,stable” OR “angina, unstable” OR “acute coronary syndrome” OR “coronary heart disease” OR “coronary atherosclerotic heart disease” OR “ischemic heart disease” OR “angina pectoris” OR “myocardial ischemic” OR “myocardial infarct*” AND Title: “sleep initiation and maintenance disorders” OR “insomnia” OR “dyssomnia” OR “sleeplessness” OR “sleep dysfunction” OR “sleep disorder*” OR “sleep quality” AND Title: “risk factor*” OR “related factor*” OR “relevant factor*” OR “influen*” OR “correlat*” OR “associat*” OR “predict*” |
| **6.Sinomed: 46 Results** |
| [( "冠心病"[标题:智能] OR "冠状动脉粥样硬化性心脏病"[标题:智能] OR "急性冠脉综合征"[标题:智能] OR "心绞痛"[标题:智能] OR "心肌缺血"[标题:智能] OR "心肌梗死"[标题:智能] OR "冠状动脉介入"[标题:智能] OR "PCI"[标题:智能] OR "胸痛"[标题:智能] OR "胸痹"[标题:智能]) AND( "失眠"[标题:智能] OR "失眠症"[标题:智能] OR "睡眠障碍"[标题:智能] OR "不寐"[标题:智能] OR "睡眠异常"[标题:智能] OR "睡眠问题"[标题:智能] OR "睡眠质量"[标题:智能]) AND( "危险因素"[标题:智能] OR "风险因素"[标题:智能] OR "影响因素"[标题:智能] OR "相关因素"[标题:智能] OR "预测因素"[标题:智能] OR "原因"[标题:智能])](javascript:toDoRelimitSearch();) |
| **7. CNKI: 269 Results** |
| （主题：冠心病 + 冠状动脉粥样硬化性心脏病 + 急性冠脉综合征 + 心绞痛 + 心肌缺血 + 心肌梗死 + 冠状动脉介入 + PCI + 胸痛 + 胸痹）AND（主题：失眠 + 失眠症 + 睡眠障碍 + 不寐 + 睡眠异常 + 睡眠问题 + 睡眠质量）AND（主题：危险因素 + 风险因素 + 影响因素 + 相关因素 + 预测因素 + 原因） |
| **8. WanFang: 958 Results** |
| ((主题=(冠心病 OR 冠状动脉粥样硬化性心脏病 OR 急性冠脉综合征 OR 心绞痛 OR 心肌缺血 OR 心肌梗死 OR 冠状动脉介入 OR PCI OR 胸痛 OR 胸痹)) AND 主题=(失眠 OR 失眠症 OR 睡眠障碍 OR 不寐 OR 睡眠异常 OR 睡眠问题 OR 睡眠质量)) AND 主题=(危险因素 OR 风险因素 OR 影响因素 OR 相关因素 OR 预测因素 OR 原因) |
| **9. VIP: 79 Results** |
| (((((((((((题名或关键词=冠心病 OR 题名或关键词=冠状动脉粥样硬化性心脏病) OR 题名或关键词=急性冠脉综合征) OR 题名或关键词=心绞痛) OR 题名或关键词=心肌缺血) OR 题名或关键词=心肌梗死) OR 题名或关键词=冠状动脉介入) OR 题名或关键词=PCI) OR 题名或关键词=胸痛) OR 题名或关键词=胸痹) AND ((((((题名或关键词=失眠 OR 题名或关键词=失眠症) OR 题名或关键词=睡眠障碍) OR 题名或关键词=不寐) OR 题名或关键词=睡眠异常) OR 题名或关键词=睡眠问题) OR 题名或关键词=睡眠质量)) AND (((((题名或关键词=危险因素 OR 题名或关键词=风险因素) OR 题名或关键词=影响因素) OR 题名或关键词=相关因素) OR 题名或关键词=预测因素) OR 题名或关键词=[原因))](https://ras.cdutcm.edu.cn:7080/s/com/cqvip/qikan/G.http/Qikan/search/index?LngMySearHistoryIdGuid=5d3bad82-5617-4857-87c8-1e1ddff41e09&from=Qikan_Article_History) |
| **10. CINAHL: 13 Results** |
| TI (“coronary artery disease” OR “coronary disease” OR “angina,stable” OR “angina, unstable” OR “acute coronary syndrome” OR “coronary heart disease” OR “coronary atherosclerotic heart disease” OR “ischemic heart disease” OR “angina pectoris” OR “myocardial ischemic” OR “myocardial infarct*”) AND TI (“sleep initiation and maintenance disorders” OR “insomnia” OR “dyssomnia” OR “sleeplessness” OR “sleep dysfunction” OR “sleep disorder*” OR “sleep quality”) AND TI (“risk factor*” OR “related factor*” OR “relevant factor*” OR “influen*” OR “correlat*” OR “associat*” OR “predict*”) |

**Supplementary File 2: Quality assessment**

**Study quality of cohort studies based on the Newcastle-Ottawa sacle**

| Study | Selection | | | | Comparability | | Outcome | | | Score | Quality of study |
| --- | --- | --- | --- | --- | --- | --- | --- | --- | --- | --- | --- |
|  | Q1 | Q2 | Q3 | Q4 | Q5 | Q5b | Q6 | Q7 | Q8 |  |  |
| Muthukrishnan2020 | 1 | 1 | 1 | 0 | 1 | 1 | 1 | 1 | 1 | 8 | High |
| Kong2021 | 1 | 1 | 1 | 0 | 1 | 1 | 1 | 1 | 1 | 8 | High |
| Huang2024 | 1 | 1 | 1 | 1 | 0 | 1 | 1 | 0 | 1 | 7 | High |
| Yakut2024 | 1 | 1 | 1 | 0 | 0 | 1 | 1 | 0 | 1 | 6 | Moderate |

Note. Q1= Representativeness of the exposed cohort; Q2 = Selection of the non exposed cohort; Q3 = Ascertainment of exposure; Q4 = Demonstration that outcome of interest was not present at start of study; Q5 = Comparability of cohorts on the basis of the design or analysis(5a= Elect the most important factor. 5b= study controls for any additional factor); Q6 = Assessment of outcome; Q7 = Was follow-up long enough for outcomes to occur; Q8 = Adequacy of follow up of cohorts.

**Study quality of case-control studies based on the Newcastle-Ottawa sacle**

| Study | Selection | | | | Comparability | | Exposure | | | Score | Quality of study |
| --- | --- | --- | --- | --- | --- | --- | --- | --- | --- | --- | --- |
|  | Q1 | Q2 | Q3 | Q4 | Q5a | Q5b | Q6 | Q7 | Q8 |  |  |
| Wassif2025 | 1 | 1 | 1 | 1 | 0 | 1 | 0 | 1 | 0 | 6 | Moderate |

Note. Q1= Is the case definition adequate; Q2 = Representativeness of the cases; Q3 = Selection of Controls; Q4 = Definition of Controls; Q5 = Comparability of cases and controls on the basis of the design or analysis(5a= Elect the most important factor. 5b= study controls for any additional factor); Q6 = Ascertainment of exposure; Q7 = Same method of ascertainment for cases and controls; Q8 = Non-Response rate.

**Study quality of cross-sectional studies based on the AHRQ**

| Study | Q1 | Q2 | Q3 | Q4 | Q5 | Q6 | Q7 | Q8 | Q9 | Q10 | Q11 | Score | Quality of study |
| --- | --- | --- | --- | --- | --- | --- | --- | --- | --- | --- | --- | --- | --- |
| Coryell 2013 | Y | Y | Y | Y | U | Y | Y | N | U | Y | U | 7 | Moderate |
| Costa2017 | Y | Y | Y | Y | U | U | Y | Y | Y | Y | U | 8 | High |
| Ning2019 | Y | Y | Y | Y | U | N | N | U | U | Y | U | 5 | Moderate |
| Cheng2022 | Y | Y | U | Y | U | N | Y | Y | U | Y | U | 6 | Moderate |
| Wu2021 | Y | Y | Y | Y | U | U | U | Y | U | Y | U | 6 | Moderate |
| Frøjd2021 | Y | Y | Y | Y | U | U | Y | Y | Y | Y | U | 8 | High |
| Zhang2021 | Y | Y | Y | U | U | N | Y | N | N | Y | U | 5 | Moderate |
| Zheng2023 | Y | Y | Y | Y | U | U | N | Y | N | Y | U | 6 | Moderate |
| Gökçe2023 | Y | Y | Y | Y | U | U | N | Y | N | Y | U | 6 | Moderate |
| He2024 | Y | Y | Y | Y | U | N | N | Y | N | Y | U | 6 | Moderate |
| Yang2024 | Y | Y | Y | Y | U | N | N | Y | U | Y | U | 6 | Moderate |
| Mo2025 | Y | Y | Y | Y | U | N | N | Y | U | Y | U | 6 | Moderate |
| Hbaieb2025 | Y | Y | Y | Y | U | Y | N | Y | N | Y | U | 7 | Moderate |
| Najfath2025 | Y | Y | Y | Y | U | N | N | Y | U | Y | U | 6 | Moderate |

Note. Q1= Define the source of information (survey, record review); Q2 = List inclusion and exclusion criteria for exposed and unexposed subjects (cases and controls) or refer to previous publications; Q3 = Indicate period used for identifying patients.; Q4 = Indicate whether or not subjects were consecutive if not population-based; Q5 = Indicate if evaluators of subjective components of the study were masked to other aspects of the status of the participants; Q6 = Describe any assessments undertaken for quality assurance purposes (e.g., test/retest of primary outcome measurements); Q7 = Explain any patient exclusions from analysis; Q8 = Describe how confounding was assessed and/or controlled; Q9 = If applicable, explain how missing data were handled in the analysis; Q10 = Summarize patient response rates and completeness of data collection; Q11 = Clarify what follow-up, if any, was expected and the percentage of patients for which incomplete data or follow-up was obtained.

N = No; U = Unclear; Y = Yes.

**Supplementary File 3: Meta-regression analysis results**


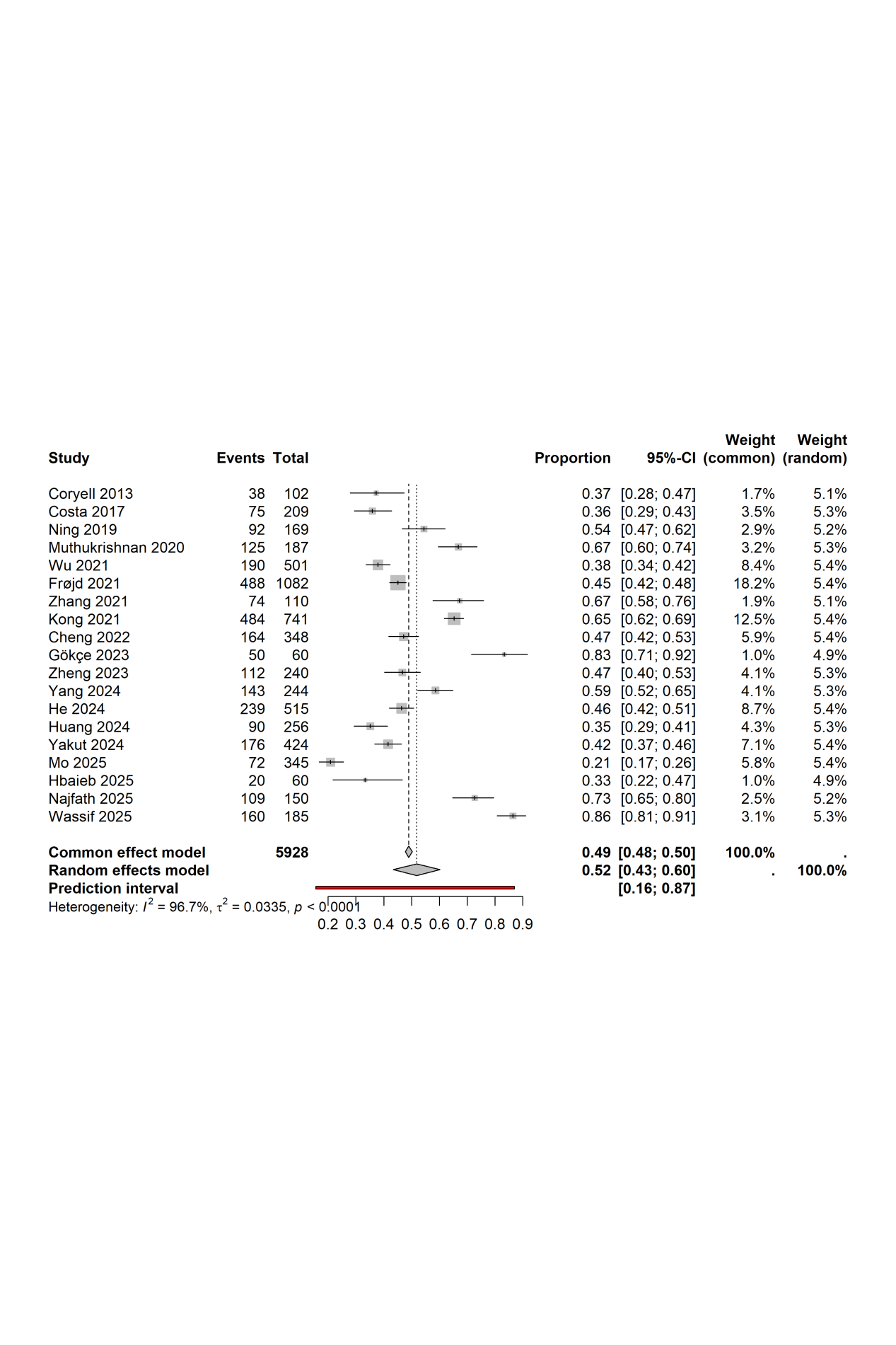


**Supplementary File 4: Meta-regression analysis results**

**Meta-regression analysis results**

| Covariate | *β* | SE | 95%CI | *P* |
| --- | --- | --- | --- | --- |
| Area | 0.872 | 0.273 | 0.444-1.714 | 0.670 |
| Publication year | 1.019 | 0.052 | 0.914-1.137 | 0.711 |
| Sample size | 1.000 | 0.001 | 0.999-1.001 | 0.537 |
| Study design | 1.011 | 0.259 | 0.581-1.758 | 0.966 |
| Diagnostic criteria | 1.174 | 0.609 | 0.383-3.599 | 0.761 |
